# Complete Genome Sequence of a Multidrug-Resistant Strain, Escherichia coli ATCC BAA-196, as a Model for Studying Induced Antibiotic Resistance Reversion

**DOI:** 10.1128/MRA.01118-19

**Published:** 2019-12-12

**Authors:** Ilya S. Korotetskiy, Monique Joubert, Setshaba Taukobong, Ardak B. Jumagaziyeva, Sergey V. Shilov, Sergey V. Shvidko, Natalya A. Suldina, Sabina T. Kenesheva, Anna Yssel, Oleg N. Reva, Aleksandr I. Ilin

**Affiliations:** a Scientific Center for Anti-Infectious Drugs, Almaty, Kazakhstan; b Centre for Bioinformatics and Computational Biology, Department of Biochemistry, Genetics, and Microbiology, University of Pretoria, Pretoria, South Africa; c Faculty of Biology and Biotechnology, al-Farabi Kazakh National University, Almaty, Kazakhstan; University of Maryland School of Medicine

## Abstract

Here, we report the complete genome sequence of the multidrug-resistant Escherichia coli strain ATCC BAA-196, a model organism used for studying possible antibiotic resistance reversion induced by FS-1, an iodine-containing complex. Two genomes, representing FS-1-treated and negative-control variants and composed of a chromosome and several plasmids, were assembled.

## ANNOUNCEMENT

Escherichia coli ATCC BAA-196 was isolated in 1988 at a chronic-care facility in Massachusetts and at first was misidentified as Klebsiella pneumoniae (1, 2). It produces extended-spectrum beta-lactamases (3, 4). This strain was used as a model of nosocomial drug-resistant infections for studying the effect of the iodine-containing complex FS-1 in reverting drug resistance (5, 6).

The strain was cultivated for 10 daily passages in Mueller-Hinton broth (HiMedia, India) with FS-1 (500 μg/ml) (FS genome) or without FS-1 (negative-control [NC] genome), in three repeats. DNA was extracted using the PureLink genomic DNA kit (Thermo Fisher). Samples were prepared according to the SMRTbell preparation guide for the PacBio RS II system. Sequencing was performed at Macrogen (South Korea) with SMRT Cell 8Pac v3 cells using the DNA polymerase binding kit P6, following the SMRTbell 20-kb library preparation protocol. For the NC and FS genomes, 334,150 and 429,631 reads, respectively, were generated (*N*_50_, 9,500 kb). After read-quality trimming using the UGENE v1.32.0 raw DNA-seq processing pipeline with default settings (7), the genomes were assembled with SMRT Link v5.0.1 with default parameters (8). The FS and NC genome assemblies are 4,682,561 and 4,682,572 bp, respectively (GC content, 51%; coverage, 250-fold); the genomes also include large plasmids of 266,396 and 279,992 bp, respectively (GC content, 47%), showing 90 to 99% sequence similarity to Klebsiella pneumoniae plasmid pKP64477b. The FS genome plasmid has an insertion of a prophage flanked by two copies of *insH* transposases. Moreover, the FS-1-treated strain contains two smaller plasmids (44,240 and 11,153 bp), which are excision products of the large plasmid. A plasmid-destabilizing effect of FS-1 was hypothesized.

**FIG 1:**
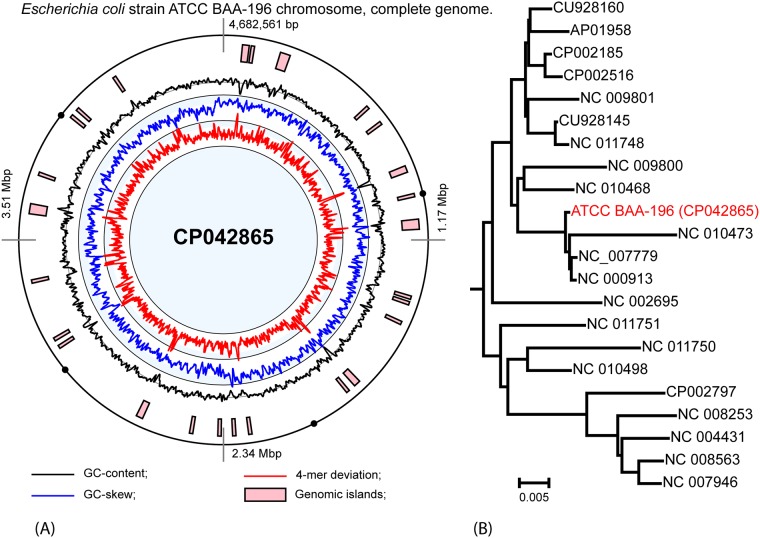
(A) Circular map of the chromosome, with predicted genomic islands and histograms of GC content, GC skew, and tetranucleotide pattern deviations calculated in an 8-kb sliding window stepping 2 kb. (B) Neighbor-joining phylogenetic tree of E. coli genomes, based on concatenated alignments of orthologous proteins.

Genomes were annotated with the RAST server (9) and manually curated. Phylogenetic inference based on concatenated alignments of 3,179 orthologous genes identified by OrthoFinder (10) (default settings) that were shared by E. coli reference genomes (Fig. 1) showed clustering of BAA-196 with the E. coli K12-related strains K12 (GenBank accession number NC_000913), K12 substrain W3110 (GenBank accession number NC_007779), and K12 substrain DH10B (GenBank accession number NC_010473).

Horizontally transferred genomic islands (Fig. 1) were identified by SeqWord Sniffer (11). Genomic islands and plasmids contain genetic determinants associated with antibiotic resistance, including beta-lactamases of the A and D classes, the tellurium resistance operon *terABCDW*, the arsenic resistance gene *arsR*, chloramphenicol and aminoglycoside acetyltransferases, and several other genes for antibiotic-modifying enzymes, drug resistance regulators, and multidrug efflux proteins. Virulence-associated fimbrial adhesin genes *ecpD* and *fimHBGFE* were acquired horizontally.

The SMRT Link DNA modification pipeline (12) was used to profile epigenetic modifications in bacterial genomes. The most abundant DNA modification was *N*^6^-adenosine methylation in both strands, at GA*T*C and GCAC(*N*^6^)G*T*T restriction sites (methylated nucleotides are underlined, and thymidine nucleotides opposing methylated ones on the complement strand are in italic type), corresponding to typical findings for E. coli DAM methyltransferases associated with EcoRV and EcoKI restriction-modification complexes (13, 14). Methylated GATC sequences often occurred in tandem with cytosine methylation in CRGKGA*T*C motifs. Two other cytosine methylation motifs, CCAGGRAH and WCCCTGGYR, controlled by EcoRII family restriction-modification genes (15) showed alternative distribution patterns in the NC and FS genomes.

### Data availability.

NCBI accession numbers are CP042865 and CP042866 for the chromosome and plasmid, respectively, of the variant NC genome (PacBio reads SRR10112463, SRR10112464, and SRR10112472) and CP042867 to CP042870 for the chromosome and three plasmids of the variant FS genome (PacBio reads SRR10112466 to SRR10112468).
