# Correction for Korotetskiy et al., “Complete Genome Sequence of a Multidrug-Resistant Strain, Escherichia coli ATCC BAA-196, as a Model for Studying Induced Antibiotic Resistance Reversion”

**DOI:** 10.1128/MRA.01291-20

**Published:** 2020-12-10

**Authors:** Ilya S. Korotetskiy, Monique Joubert, Setshaba Taukobong, Ardak B. Jumagaziyeva, Sergey V. Shilov, Sergey V. Shvidko, Natalya A. Suldina, Sabina T. Kenesheva, Anna Yssel, Oleg N. Reva, Aleksandr I. Ilin

**Affiliations:** a Scientific Center for Anti-Infectious Drugs, Almaty, Kazakhstan; b Centre for Bioinformatics and Computational Biology, Department of Biochemistry, Genetics, and Microbiology, University of Pretoria, Pretoria, South Africa; c Faculty of Biology and Biotechnology, al-Farabi Kazakh National University, Almaty, Kazakhstan

## AUTHOR CORRECTION

Volume 8, no. 50, e01118-19, 2019, https://doi.org/10.1128/MRA.01118-19.

The history of the strain Escherichia coli ATCC BAA-196 was misinterpreted. This strain was laboratory designed by transferring antibiotic resistance plasmid pMG223 obtained from Klebsiella pneumoniae ATCC 14714 to E. coli J53-2 (G. A. Jacoby and L. Sutton, Antimicrob Agents Chemother 35:164−169, 1991, https://doi.org/10.1128/AAC.35.1.164).

